# Efficacy, Toxicity, and Prognostic Factors of Re-treatment With [177Lu]Lu-DOTA-TATE in Patients With Progressing Neuroendocrine Tumors: The Experience of a Single Center

**DOI:** 10.7759/cureus.47506

**Published:** 2023-10-23

**Authors:** Maria Manuel Silva, Marta Canha, Daniela Salazar, João Sergio Neves, Gonçalo Ferreira, Davide Carvalho, Hugo Duarte

**Affiliations:** 1 Endocrinology, Diabetes and Metabolism, Centro Hospitalar Universitário de São João, Porto, PRT; 2 Nuclear Medicine, Instituto Português de Oncologia Porto, Porto, PRT

**Keywords:** [177lu]lu-dota-tate, net, neuroendocrine tumour, prrt, peptide receptor radionuclide therapy

## Abstract

Purpose: Peptide receptor radionuclide therapy (PRRT) is an effective and safe treatment of unresectable or metastatic, progressive neuroendocrine tumours (NETs). However, if progression occurs after the initial PRRT, treatment options remain limited.

Our aim was to evaluate the efficacy and safety of a repeat ^177^Lutetium-[DOTA°,Tyr^3^]octreotate ([^177^Lu]Lu-DOTA-TATE) PRRT course in patients with progressive NET after the first [^177^Lu]Lu-DOTA-TATE PRRT (peptide receptor radionuclide therapy first treatment (PRRT1)).

Methods: This is a nine-year retrospective observational study of 20 patients who were re-treated with PRRT (peptide receptor radionuclide therapy retreatment (PRRTR)) after PRRT1.

Results: The median progression-free survival (PFS) following PRRT1 was 32 months (interquartile range (IQR): 16.5-44.5). After PRRT1, all 20 patients progressed. Of the 20 patients included, two were lost during follow-up. The median PFS after PRRTR was 17.5 months (IQR: 7-39). At the time of analysis, 15/18 patients progressed, and 3/18 had stable disease after PRRTR. Among those patients who progressed, the median time to progression was nine months (IQR: 0-17). The median overall survival from the time of the first cycle of PRRT1 was 66 months (IQR: 65-90). No significant renal or liver toxicity was reported, nor was there a drop in haemoglobin. The decrease in platelet count after PRRTR was statistically significant (p=0.03). Two cycles at PRRTR (vs. 1) were associated with a longer PFS (p=0.014) and the presence of metastases pre-PRRTR was associated with a shorter time to progression following PRRTR (p=0.04).

Conclusion: Patients who progressed after PRRT1 can achieve good PFS and minor toxicity. Our study reinforces the efficacy and safety of PRRTR and provides an analysis of factors associated with better outcomes, which can aid clinicians in clinical decision-making.

## Introduction

Neuroendocrine neoplasms (NENs) are a heterogeneous group of neoplasms that arise in scattered endocrine cells of the diffuse neuroendocrine system. Neuroendocrine neoplasms can originate from different organs, although most do so from the gastroenteropancreatic system and lung [1-3].

The all-embracing expression of somatostatin receptors (SSTRs) by NENs can be used for diagnosis and treatment purposes. In terms of diagnosis, somatostatin receptor imaging (SRI) with [^111^In]In-pentetreotide along with single photon emission computed tomography (OctreoScan®) has been extensively used. However, recently, imaging with [^68^Ga]Gallium-DOTA somatostatin analogues (^68^Ga-SSA) along with positron emission tomography/computed tomography (PET/CT) is being increasingly utilized. This modality has better diagnostic performance, exposes the patient to less radiation, and has a better spatial resolution [3,4]. In terms of treatment, activated SSTRs have anti-secretory and anti-proliferative activity and are targeted by stable somatostatin analogues (SSAs) for the treatment of functional syndromes and for reducing tumour growth in NENs. In cases where beta- and/or gamma-emitting radionuclides, such as yttrium-90 (^90^Y) and/or lutetium-177 (^177^Lu), are in evidence together with stable SSAs, this permits the internal radiation of SSTR-expressing NENs by peptide receptor radionuclide therapy (PRRT) [2,4]. The most commonly used SSTR compounds are DOTA-TATE and DOTA-NOC [5].

Treatment options are limited in the case of metastatic and advanced neuroendocrine tumours (NETs). The only curative treatment available is surgery, which is only feasible for less than 10% of patients. Nevertheless, several systemic treatment options can provide symptomatic benefit and prolong survival, including SSAs, chemotherapy, molecular targeted treatments (everolimus and sunitinib), alpha-interferon, and PRRT [2-4].

The efficacy of PRRT was proven in a 2017 multicenter randomised controlled trial, the Neuroendocrine Tumors Therapy (NETTER-1) trial [6]. This study evaluated the efficacy and safety of [^177^Lu]Lu-DOTA-TATE (compared with high-dose octreotide long-acting release (LAR)) in patients with advanced SSTR-positive midgut NETs who had progressed on octreotide LAR. The study demonstrated a significant tumour response rate of 18% in the PRRT group vs. 3% in the control, with a 79% reduction in the risk of disease progression or death. Peptide receptor radionuclide therapy achieved progression-free survival (PFS) of 65.2% at month 20 (95% confidence interval (CI); 50.0-76.8) vs. 10.8% (95% CI; 3.5-23.0)) [6]. Following these results, [^177^Lu]Lu-DOTA-TATE was approved by the US Food and Drug Administration in 2018 and the European Medicines Agency in 2017 for the treatment of adults with SSTR-positive gastroenteropancreatic NETs (GEP-NETs) [7]. Currently, the most widely used is PRRT with [^177^Lu]Lu-DOTA-TATE [4].

Peptide receptor radionuclide therapy is usually well tolerated, providing a clinically significant improvement in quality of life. The most common side effects are nausea and/or vomiting. Carcinoid crises (<1% of patients), bone marrow suppression, and hepatic and renal toxicity are rare, and the development of myelodysplastic syndrome or acute myeloid leukaemia occurs in only 1% to 2% of cases. Yttrium-90 [^90^Y] has a more energetic beta particle, and higher amounts of bone marrow/renal toxicity have been described [4,8-10].

Although PRRT is an effective treatment, patients with metastatic disease invariably progress over time. This has led to research into the potential practice of re-treatment with PRRT, or salvage PRRT, in patients with progressive disease after initial [^177^Lu] and/or [^90^Y] -based PRRT [5,11,12]. A recent meta-analysis suggests that re-treatment with [^177^Lu] based PRRT provided encouraging median PFS in patients with NETs with a safety profile similar to initial PRRT [5,11,12].

The main aim of this study is to evaluate the efficacy and toxicity of a repeat PRRT course in patients with progressive disease after initial [^177^Lu]Lu-DOTA-TATE and to identify potential predictors of response.

This article was previously presented as a meeting abstract at the X Advanced Course of Endocrinology on April 8, 2022.

## Materials and methods

Study design and included population

This is a nine-year retrospective observational study conducted in a population of patients submitted to PRRTR from January 2011 to December 2020 at the Department of Nuclear Medicine, Instituto Português de Oncologia Porto, Portugal. Patients with well-differentiated NET of gastro-entero-pancreatic, thoracic, or pelvic origin who had received peptide receptor radionuclide therapy retreatment (PRRTR) with [^177^Lu]Lu-DOTA-TATE were eligible for inclusion. Peptide receptor radionuclide therapy is defined as being a second treatment with [^177^Lu]Lu-DOTA-TATE after a period of disease control following peptide receptor radionuclide therapy first treatment (PRRT1) and subsequent disease progression. The only patients excluded were those without progression data for PRRTR.

The retrospective analysis was performed in compliance with the principles of the Declaration of Helsinki and its amendments. Ethics approval is not required for this type of study, as this was a retrospective analysis of patient data and no identifiable patient data were provided. For this type of study, formal consent is not required, in accordance with the national legislation and the institutional requirements.

Criteria for PRRT and treatment protocol

Criteria for PRRT (PRRT1 and PRRTR) included non-operable or metastatic neuroendocrine tumours with sufficient tracer uptake on somatostatin receptor imaging (SRI)(i.e., uptake in tumour tissue higher than the normal uptake by the liver, Krenning score ≥ 2) obtained three weeks before the start of PRRT and also evidence of progressive disease, according to response evaluation in solid tumours with CT, magnetic resonance imaging (MRI), or SRI ([^68^Ga]-DOTANOC PET/CT). In addition, patients required an adequate functional bone marrow reserve (European Neuroendocrine Tumour Society guidelines [9]) and normal renal function (estimated glomerular filtration rate (eGFR) >30 ml per minute per 1.73 m^2^).

All PRRT treatments were [^177^Lu]Lu-DOTA-TATE. Long-acting SSAs were stopped at least four weeks prior to treatment, and short-acting SSAs at least 24 hours in advance. For kidney protection, an intravenous infusion (containing 25g of both lysine and arginine in saline) was infused over four to six hours, starting 30 minutes before the administration of the radiopeptide. Administration of the radiopharmaceutical was performed over a period of approximately 30 minutes. The administered dose per cycle varied according to the date of administration. Prior to 2015, the department protocol advocated a dose of 5.5 GBq per cycle; however, since 2015, the administered dose has been approximately 7.4 GBq per cycle. No personalised dosimetry was applied for PRRT1 or PRRTR. At PRRT1, if no contraindications were identified, the interval between cycles was six to 12 weeks, up to a maximum of four cycles. The PRRTR protocol included two cycles.

Routine blood analysis for haematology, liver, and kidney function was performed four to six weeks after each cycle.

Significant renal impairment, haematological changes, and clinical deterioration prompted a reevaluation of the indication to proceed with further cycles. The number of PRRT cycles was dependent on the patient’s clinical condition, tumour load, and recorded toxicities.

After completing PRRT, patients were followed up at three- to six-month intervals. If disease progression was identified, then a multidisciplinary team discussed the consideration of other treatments or PRRTR. To be eligible for PRRTR, patients required a PFS of ≥6.0 months from the last cycle of PRRT1 and had to comply with the criteria previously required for PRRT1.

Data collection and response assessment

Data regarding patients and diseases were collected from hospital electronic records and imaging. The PFS was calculated from the date of the start of PRRT up to the date of progression. Progression was defined as either clinical (i.e., worsening symptoms or general deterioration) or radiological (i.e., either at mid- or end-of-treatment restaging) progression of the disease or even death.

In those patients who had not progressed, the date of the most recent imaging showing stable disease (SD) was recorded and used in the statistical analysis.

Peptide receptor radionuclide therapy-related toxicity was identified based on the routine blood analysis for haematology, liver, and kidney function pre- and post-treatment. This was defined in accordance with the Common Toxicity Criteria for Adverse Events (CTCAE), Version 5 [13].

Statistical analysis

Continuous variables are described as mean ± standard deviation or median (25th-75th percentiles), and categorical variables as proportions (percentages). Linear and logistic regressions were used to evaluate the associations of clinical variables with a better outcome. Kaplan-Meier (K-M) survival plots of time to progression were performed. T-tests were used to assess toxicity through the comparison of several biochemical parameters. All analyses were conducted with the statistical software package Stata IC, Version 14.2 (StataCorp LLC, College Station, TX), using a dataset obtained from the National Institute of Diabetes and Digestive and Kidney Diseases (NIDDK) Data Repository. A two-sided p-value < 0.05 was considered to be statistically significant.

## Results

Baseline population characteristics

Patient and tumour characteristics are shown in Table 1.

**Table 1:** Patient and tumour characteristics SSA: somatostatin analogues; PRRTR: peptide receptor radionuclide therapy retreatment

Characteristic	_
Sex, n (%)	
Female	6 (30.0%)
Male	14 (70.0%)
Age at diagnosis (years), mean (SD)	53.3 (12.4)
Tumour location, n (%)	
Small intestine	7 (35.0%)
Colon	1 (5.0%)
Rectum	2 (10.0%)
Pancreas	5 (25.0%)
Lung	2 (10.0%)
Pelvis	2 (10.0%)
Ovaries	1 (5.0%)
Tumour size	
< 1cm, n (%)	0
1-2 cm, n (%)	4 (20.0%)
2-3 cm, n (%)	2 (10.0%)
>3 cm, n (%)	3 (15.0%)
Unavailable, n (%)	10 (50.0%)
Tumour grade	
Grade 1, n (%)	8 (40.0%)
Grade 2, n (%)	7 (35.0%)
Unavailable, n (%)	5 (25.0%)
Metastasis at diagnosis	
Yes, n (%)	13 (65.0%)
No, n (%)	6 (30.0%)
Unknown	1 (5.0%)
Metastasis previous PRRTR, n (%)	18 (90.0%)
Lung, n (%)	2 (10.0%)
Ganglionar, n (%)	15 (75.0%)
Bone, n (%)	10 (50.0%)
Liver, n (%)	18 (90.0%)
Previous treatment	
Surgery, n (%)	13 (65.0%)
SSAs, n (%)	19 (95.0%)
Chemotherapy, n (%)	9 (45.0%)
Radiotherapy, n (%)	3 (15.0%)
Liver targeted treatment, n (%)	8 (40.0%)
Interferon/sunitinib/everolimus, n (%)	3 (15.0%)

The baseline population at this point comprised 20 patients, of which 70% were male, with an overall average age of 53.3 ± 12.4 at diagnosis. Most patients had small intestinal NET, with pancreatic NET being the second most prevalent. Three of the 20 tumours were functional. The presence of metastasis was documented at both the diagnosis and prior to the initiation of PRRTR. Sixty-five percent of patients had metastasis at diagnosis, whereas before PRRTR, the majority of patients (90%) had metastasis, reflecting the progression of the disease over time.

Peptide receptor radionuclide therapy first treatment (PRRT1)

**Figure 1:**
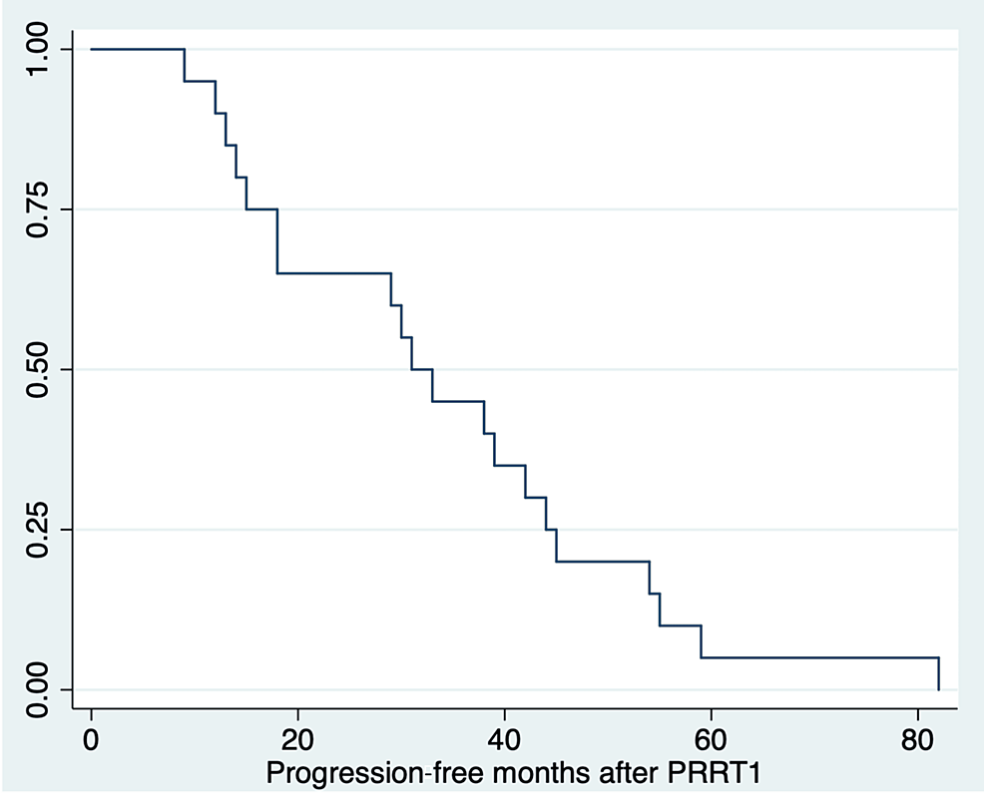
Kaplan-Meier curve for PFS after PRRT1 (median PFS: 32 months, IQR: 16.5–44.5 months) PRRT1: peptide receptor radionuclide therapy first treatment

The median cumulative administered dose was 17.2 GBq (range 14.1-29.6 GBq). The majority of patients (19/20 of the patients) had three cycles of [^177^Lu]Lu-DOTA-TATE and one patient had four cycles. The median administered dose per cycle was 5.7 GBq (range: 4.7-7.3). After PTRT1, all 20 patients progressed. The median PFS following PRRT1 was 32 months (interquartile range (IQR): 16.5-44.5 months), and the minimum PFS was 9 months. Figure 1 shows the K-M curve for PFS at PRRT1.

The median follow-up time was 102.0 months (IQR: 2-144 months) from the start of PPRT1.

Peptide receptor radionuclide therapy retreatment (PRRTR)

**Figure 2:**
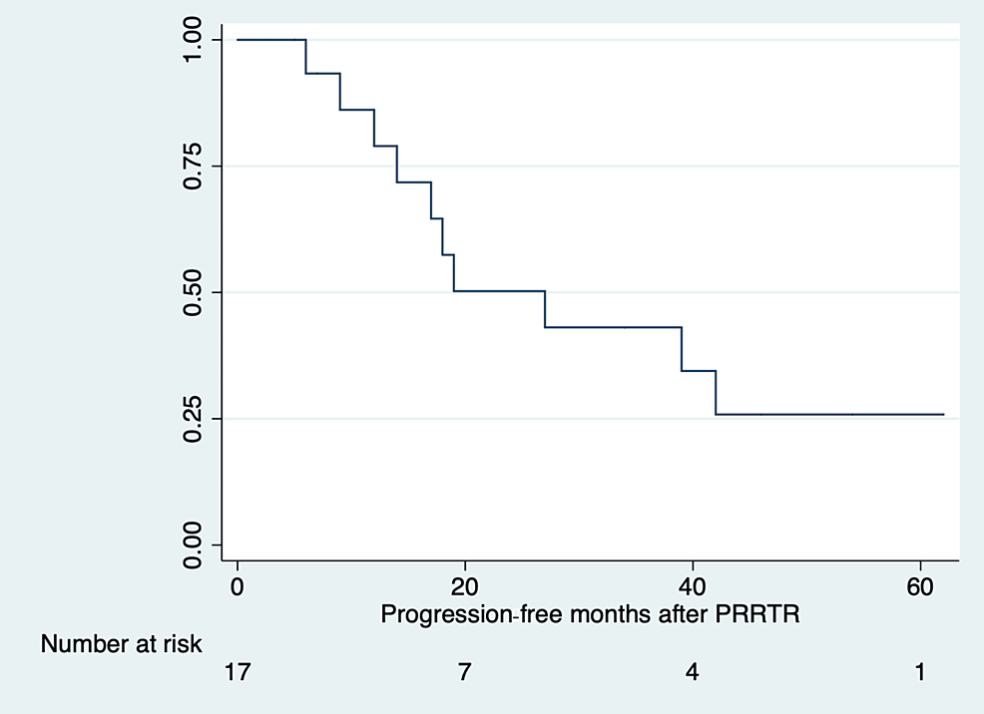
Kaplan-Meier curve for PFS after PRRTR (median PFS: 17.5 months, IQR: 7–39 months) PRRTR: peptide receptor radionuclide therapy retreatment

Of the 20 patients originally included in our study, two were lost during follow-up. The average time between the initiation of PRRT1 and PRRTR was 42 months (range: two to 91 months). The median cumulative administered dose was 12.2 GBq (range: 4.8-14.8 GBq), resulting in total (PRRT1+PRRTR) median cumulative administered doses of 29.2 GBq (range: 24.0-37.0 GBq). At PRRTR, the median of cycles administered was two (range: 1-2, mean 1.7). The efficacy of PRRTR was evaluated in 18 patients. In terms of PFS, the median PFS (n=18) after PRRTR was 17.5 months (IQR: 7-39 months). Figure 2 shows the K-M curve for PFS at PRRTR.

At the time of analysis, 83.3% of patients had progressed. Among those patients who progressed, the median time to progression was nine months (IQR: 0-17 months). All patients had survival data, and 13 out of 20 (65%) died. Among those who died, the cause of six out of 13 was disease progression. Regarding the overall survival (OS) analysis, the median OS from the time of the first cycle of PRRT1 was 66 months (IQR: 56-90 months).

Variables associated with PRRTR

Table 2 shows the analysis of the variables associated with PFS at PRRTR, time to progression after PRRTR, and status at follow-up.

**Table 2:** Analysis of factors associated with PFS at PRRTR, time to progression after PRRTR, and status at follow-up PFS: progression-free survival; PRRTR: peptide receptor radionuclide therapy retreatment; CI: confidence interval: SSA: somatostatin analogues; no.: number: PRRT1: peptide receptor radionuclide therapy first treatment

	PFS at PRRTR				Time to progression after PRRTR				Status at follow-up		
Variable (n=18)	β	95% CI	p-value		β	95% CI	p-value		β	95% CI	p-value
Age at diagnosis, years	-0.373	-1.145 − 0.400	0.322		-0.315	-0.808 − 0.178	0.191		0.050	-0.030 − 0.131	0.221
Sex, female (vs. male)	1.508	-20.011 − 23.027	0.884		-3.833	-18.549 − 10.882	0.583		1.322	-1.072 − 3.716	0.279
Metastasis at diagnosis	-12.585	-33.059 − 7.894	0.211		-7.750	-20.397 − 4.897	0.208		1.897	-0.235 − 4.029	0.081
Metastasis previous PRRTR	-15.625	-45.177 − 13.927	0.279		-27.786	-44.910 − -10.661	0.004		0.693	-2.247 − 3.633	0.644
Previous treatments											
SSAs	-11.529	-53.191 − 30.132	0.566		3.286	-20.517 − 27.088	0.770		-0.684	-0.345 − 1.714	0.995
Resection of primary	-12.442	-31.095 − 6.212	0.177		-10.167	-20.692 − 0.359	0.057		0.523	-1.381 − 2.428	0.590
Chemotherapy	6.550	-12.548 − 25.648	0.478		2.389	-9.687 − 14.465	0.676		-1.609	-3.642 − 0.4227	0.121
Radiotherapy	-1.250	-31.933 − 29.433	0.932		11.714	-11.115 − 34.543	0.288		0.182	-2.423 − 2.787	0.891
Liver targeted treatments	-10.468	-29.459 − 8.524	0.260		-4.900	-17.192 − 7.392	0.405		0.916	-1.076 − 2.909	0.367
Interferon/sunitinib/everolimus	-13.625	-43.454 − 16.204	0.347		-10.786	-33.778 − 12.207	0.329		0.182	-2.423 − 2.787	0.891
PRRT1											
No. of cycles (4 vs. 3)	-18.118	-59.115 − 22.880	0.363		-10.786	-33.778 − 12.207	0.329		0.368	-0.699 − 1.437	0.478
Cumulative dose	0.001	-0.004 − 0.001	0.323		0.0003	-0.002 - 0.001	0.730		0.00007	-0.0002 − 0.0004	0.651
PFS after PRRT1	-0.391	-0.863 − 0.080	0.098		-0.285	-0.671 − 0.102	0.136		-0.030	-0.082 − 0.022	0.266
PRRTR											
No. of cycles (2 vs. 1)	23.138	5.437 - 40.840	0.014		8.833	-5.089 − 22.755	0.194		-1.322	-3.716 − 1.072	0.279
Cumulative dose	0.002	-0.001 - 0.005	0.095		0.0006	-0.001 - 0.002	0.505		-0.001	-0.002 − 3.270	0.051

Concerning PFS after PRRTR, patients submitted to two cycles of PRRTR had more time until progression compared to those treated with just one cycle (p=0.014). Patients with metastasis from previous PRRTR had fewer months without progression (p=0.004). With regards to status at follow-up, no factor reached statistical significance.

Peptide receptor radionuclide therapy retreatment toxicity analysis

Table 3 describes a toxicity analysis according to CTCAE, Version 5 [13], for both previously and after PRRTR.

**Table 3:** Toxicity analysis in blood count and creatinine according to the Common Toxicity Criteria Adverse Events, version 5 PRRTR: peptide receptor radionuclide therapy retreatment [13]

		Previous PRRTR (n=20)				After PRRTR (n=18)				
Toxicity		Grade1	Grade 2	Grade 3		Grade 1	Grade 2	Grade 3		p-value
Haemoglobin		3	0	1		1	1	1		1.000
Leucocytes		0	0	0		2	2	0		0.054
Platelets		4	0	0		10	0	0		0.055
Creatinine		10	2	0		8	2	1		0.331

Prior to PRRTR, four patients had anaemia (three grade 1, one grade 3), four patients had thrombocytopenia (four grade I), and there was no case of leucopenia. One patient received one cycle of PRRTR, despite having grade 3 anaemia, due to the progression of the disease and a lack of treatment alternatives. Red cell concentrate was administered prior to PRRTR. After PRRTR, three patients had anaemia (one grade I, one grade II, one grade III), four patients had leucopenia (two grade I, two grade II), and 10 patients had grade I thrombocytopenia. Leucopenia toxicity analysis according to CTCAE did not attain statistical significance; however, a tendency to significance (p=0.054) was evident, which was also true for the thrombocytopenia toxicity analysis (p=0.055). A comparison of the mean platelet count pre- and post-PRRTR (Table 3) showed a significant drop after PRRTR (p=0.03).

There was no evidence of a significant drop in red and white blood cell counts in this analysis. One patient had grade 3 PRRTR-related nephrotoxicity; however, renal toxicity post-PRRTR was not statistically significant (Tables 3, 4).

**Table 4:** Analytical evaluation before and after PRRTR PRRTR: peptide receptor radionuclide therapy retreatment; WBC: white blood count; AST: aspartate aminotransferase; ALT: alanine transaminase; GGT: gamma-glutamyl transferase; eGFR: estimated glomerular filtration rate

Variable	Before PRRTR	After PRRTR	p-value
_	(n=18)	(n=18)	_
Hemoglobin (g/dL), mean (SD)	13.228 (2.387)	13.061 (2.393)	0.492
WBC (x10^9/L), mean (SD)	7.257 (2.807)	6.394 (3.484)	0.368
Platelets (x10^9/L), mean (SD)	250.778 (89.691)	211.055 (90.006)	0.029
AST (U/L), mean (SD)	25.059 (9.516)	25.353 (9.076)	0.841
ALT (U/L), mean (SD)	24.882 (13.421)	22.471 (8.740)	0.263
GGT (U/L), mean (SD)	88.176 (99.958)	87.588 (106.831)	0.936
Total bilirubin (μmol/L), mean (SD)	12.376 (7.105)	14.303 (12.028)	0.194
Albumin (g/L), mean (SD)	42.027 (2.841)	41.940 (3.737)	0.906
Creatinine (mg/dL), mean (SD)	0.981 (0.228)	1.010 (0.312)	0.598
eGFR (%), mean (SD)	83.833 (23.241)	83.611 (23.228)	0.942

## Discussion

This research concerns a retrospective longitudinal study of patients who had been treated with [^177^Lu]Lu-DOTA-TATE and, following disease progression, had been re-treated with a second course of [^177^Lu]Lu-DOTA-TATE. The study has four main findings. First, patients who progressed after the first course of PRRT can achieve a reasonable PFS with PRRTR. Second, patients treated with two cycles at PRRTR had longer PFS at PRRTR when compared to patients treated with one cycle. Third, patients without metastasis before PRRTR registered more time until progression. Fourth, mild thrombocytopenia was the only statistically significant toxicity found.

A systematic literature review and meta-analysis were carried out in 2021 to assess the efficacy and safety of ^177^Lu-DOTATATE re-treatment in patients with advanced NETs [11]. The pooled median PFS in those patients who received both initial PRRT and re-treatment with [^177^Lu]Lu-DOTA-TATE (n=3 studies with 211 patients) was estimated to be 14.35 months (CI 95% (12.35-16.35 months)). To diminish statistical heterogeneity, two studies were initially removed from this evaluation, namely, Rudisile et al. [14] (heavily pre-treated patients) and Severi et al. [15] (suboptimal dose). The three studies that were included were Sabet et al. (n= 33 patients, median PFS of 13 months) [16], Limouris et al. (n= 13 patients, median PFS of 20.0 months) [17], and Van der Zwan et al. (n=168 patients, median PFS of 14.6 months) [12]. This latter study is the largest cohort that has been published, with a median follow-up time of 88.6 months for a total of 168 patients with progressive bronchial NET or GEP-NET, with a PFS at PRRT1 ≥18 months required to PRRTR and PFS at PRRT1 being significantly associated with PFS at PRRTR. Sabet et al. had previously demonstrated this phenomenon, showing that PFS after initial PRRT was correlated with PFS after PRRTR, with patients with a history of durable PFS after initial PRRT tending to have long-lasting PFS after re-treatment [16]. In our study, a minimal PFS at PRRT1 ≥6 months was necessary for re-treatment eligibility. In fact, although this required PFS, the median PFS at PRRT1 was 32 months. The PFS at PRRT1 was close to that of Limouris et al. (34.3 months) and Van der Zwan et al. (35.4 months) and was superior to Sabet et al. (22 months) [12,16,17]. In our study, a median PFS at PRRTR of 17.5 months with a median follow-up time of 102.0 months (IQR (72-144 months)) was demonstrated. Only Limouris et al. recorded a superior PFS at PRRTR [17].

Concerning OS data assessment, the median OS from the time of the first cycle of PRRT1 was 66 months. The OS was mentioned in three studies. Van der Zwan et al. demonstrated a median OS of 80.8 months, and the other two studies were those of Rudisile et al. (mean OS of 105 months) and Severi et al. (median OS of 63.8 months) [12, 14-15].

When we compared the cumulative administered doses (PRRT1 + PRRTR) with previous studies, the median cumulative administered dose was lower compared to the previous studies (29.2 GBq (24.0-37.0 GBq) vs. 44.7 GBq (26.3-46.4 GBq) [12], 44.3 GBq (30.0-83.7 GBq) [16], and 44.0 GBq (33.5-47.0 GBq) [14]. This lower median was the result of a lower dose being administered at PRRT1. However, the administered dose at PRRTR was similar to those used in other studies [12,16]. This lower cumulative administered dose was not reflected in a lower level of efficacy.

We analysed factors associated with PFS at PRRTR and also with time to progression after PRRTR. Although these assessments may seem ambiguous, we chose to do so on account of the limited sample size in order to increase statistical power. Patients treated with two cycles (vs. one) at PRRTR had longer PFS. The PRRTR protocol consisted of two cycles wherever possible. Five patients had one cycle of PRRTR, two of them due to grade 3 adverse events (anaemia and renal toxicity), and the rest owing to bad functional status or refusal by the patient. In addition, patients with metastasis before PRRTR recorded less time to progression. Studies with analyses of factors related to PRRTR response are limited. For instance, Vaughn et al. [5] analysed 47 patients with a combination of PRRT with [^90^Y]Y-DOTA-TATE and [^177^Lu]Lu-DOTA-TATE and demonstrated that male gender and a higher proportion of liver metastases were associated with reduced PFS post-PRRTR. In our study, 90% of patients had liver metastasis prior to PRRTR, which could have contributed to less time to progression.

In terms of safety and toxicity, significant haematotoxicity was only observed in one patient (5.6%). This patient had grade 3 anaemia post-PRRTR. However, when haemoglobin prior to PRRTR was analysed, grade 3 anaemia was already present. The patient in question had a pancreatic NET and was submitted to chemotherapy and three cycles of PRRT1, with a PFS of nine months (the lowest PFS of the entire population under study), and one cycle of PRRTR, with a PFS of 12 months. In fact, haemoglobin post-PRRTR was 1 g/dL superior to pre-PRRTR. Concerning nephrotoxicity, one patient had grade 3 nephrotoxicity; however, renal toxicity post-PRRTR was not statistically significant. According to CTCAE criteria [13], haemoglobin and leucocyte toxicity were not statistically significant. Ten patients had grade I thrombocytopenia post-PRRPR (vs. four pre-PRRTR) with a tendency towards statistical significance (p=0.055). A statistically significant drop in mean platelets was observed post-PRRTR. However, thrombocytopenia was mild, with a mean value of 211x10^9/L platelets. The myelodysplastic syndrome was not observed in any of the patients in our study, despite the long follow-up period. Lower adverse event incidence can be explained by lower cumulative doses when compared with other studies [12,14]. Nevertheless, Van der Zwan et al. showed that there was no indication of increased renal or haematological toxicity after the administration of higher cumulative doses of [^177^Lu]Lu-DOTA-TATE than those administered during initial PRRT.

Our study contains limitations that need to be addressed, the major ones being the limited number of patients included and the retrospective setting of the study. The small number of patients could be underpowered to detect statistically significant differences.

## Conclusions

The current literature on PRRTR is scarce and mainly includes studies with different combinations of radiolabeled somatostatin analogues. Studies with [^177^Lu-DOTA,Tyr3]octreotate-based initial and re-treatment PRRT are even rarer. This study showed that patients who progressed after PRRT1 can achieve good PFS and minor toxicity after PRRTR. In sum, this study contributes to scientific knowledge as it reinforces the efficacy and safety of this treatment and provides an analysis of those factors associated with better outcomes at PRRTR, which can aid clinicians in clinical decision-making.
